# Alcohol brings burdens: A global and continent wise study on alcohol consumption and global burden of diseases

**DOI:** 10.1371/journal.pone.0270998

**Published:** 2022-07-28

**Authors:** Ruwan Jayathilaka, Oshada Athukorala, Sanduni Ishara, Dishani Silva, Tanya Pathirage

**Affiliations:** 1 Department of Information Management, SLIIT Business School, Sri Lanka Institute of Information Technology, Malabe, Sri Lanka; 2 SLIIT Business School, Sri Lanka Institute of Information Technology, Malabe, Sri Lanka; Chung Shan Medical University, TAIWAN

## Abstract

This article investigates alcohol consumption attributable burden of diseases. The present study considers the overall effect of Human Development Index (HDI), Socio Demographic Index (SDI) and Gross Domestic Product (GDP) for income to measure how these variables impact Global Burden of Diseases (GDB), bringing a different perspective to the results. Data from over 177 countries mainly including European, Asian, North American, South American, African and Australian regions were analysed from 2000 to 2019. A Panel regression technique was applied, and Fixed Effects (FE) and Random Effects (RE) estimations were chosen to derive outcomes of the Hausman test. The findings reflected that alcohol consumption (wine, beer, spirit and other alcohol) has a positive significant relationship with the Global Burden of Diseases (GBD) globally and in the African continent excluding North America and South America. Human Development Index (HDI) have a negative impact on GBD on all countries. Particularly HDI have a negative impact on GBD in African continent except other continents. Also, in the African continent and globally, GBD has a significant positive and in Europe, a significant negative impact on the Socio Demographic Index (SDI). The findings demonstrate the overall picture of the impact of alcohol consumption and other variables on GBD and provide suggestions on how these variables should be tackled in the future to reduce GBD. This is the initial empirical study that investigates the impact of alcohol consumption, analysing the combined effect of HDI, SDI and income on the GBD of continent wise and globally.

## Introduction

Liquor is devoured around the world at numerous events at social gatherings and occasions. People are delighted by drinking socially and moderately from wine to beer and spirit. Since the year 2000, more increased alcohol consumption globally is observed except the World Health Organizations (WHO) European region which stands the same where current drinkers are estimated to pass more than 2.3 billion worldwide [1]. Reflecting on current ratings of alcohol consumption, objectives for decreasing misuse of alcohol at the global level are difficult to be accomplished [2].

With changes in human’s perception towards alcohol, it can be inferred that the burden on illness attributable to alcohol has expanded. Lately, according to significant discoveries, more than half of the population in Europe, America and Western Pacific districts consume liquor and even young adolescents below 15 years of age are used to liquor as a substance [1]. These kinds of key discoveries probably have given rise to more deliberations about the subject of liquor utilisation and the burden of illnesses where most suggestions aim to devise approaches and charges concerning the negative consequences of liquor consumption. Nowadays, alcoholic beverages have ended up being a more common substance consumed in everyday schedules of populaces in many social orders and accessible anytime universally. Improvement of culture, economy, executed and upheld alcohol related policies and easy access to alcoholic beverages are the impressive components that influence liquor consumption [1].

Global Burden of Diseases (GBD) is a tool that gives full view of what disables and kills human across the countries, age, time and sex, expressed in health loss from hundreds of diseases, injuries, and risk factors in terms of quantity. This measurement is beneficial for the health care framework and it provides examined insights into this type of information that can assist in making choices to overcome certain harms that are caused [3]. GBD gives information over 195 nations and regions for more than 350 illnesses from the year 1990 onwards permitting comparison over time [3]. Alcohol utilisation may be a hazard substantially impacting on the burden of diseases [4]. Regarding alcohol consumption levels, findings are unavailable to confirm that alcohol is attributable for decreased health loss [5]. Approximately 7.2% of males and 2.2% of females are being influenced individually by GBD for misusing liquor [1]. Over 300 diseases and harmful conditions were identified by the WHO due to irresponsible alcohol consumption patterns [1].

The main objective of this study is to investigate the long-term relationship between global alcohol consumption and GBD between the years 2000 and 2019 continent wise and globally. A deep study of the correlation between alcohol consumption and GBD within the above mentioned period, and how the global intention of sustainable development goal in reducing global alcohol consumption will be discussed subsequently. This study has been conducted due to two specific reasons. First, this research study covers 177 countries worldwide including all five continents using recent data collected over the past 20 years. Hence, the study is comprehensive (wide coverage of countries and to the best of the researcher’s knowledge, so far studies have not focussed on this topic under the variables selected in the present study) and timely. In addition, a separate analysis is conducted for each continent with the global level analysis which further enhances the significance of the study. The second reason is that the variables selected for the analysis are unique compared to those considered in previous studies. In contrast, education, poverty and other major measurements which count the level of human development were considered as variables in prior studies.

The present study considers the overall effect of Human Development Index (HDI), Socio Demographic Index (SDI) and Gross Domestic Product (GDP) for income to measure how these variables impact GDB, bringing a different perspective to the results. As such, in a nutshell, considering the above mentioned variables give insights into the real and holistic situation globally as well as the situation continent wise.

This article contains the introduction in the first section which describes the background study of this research. Then, the second chapter explains the literature review and contains a synopsis of relevant literature. Data and methodology are detailed in the third chapter with details of summary statistics for key variables and then the relationship is discussed between the variables like beer, wine, spirit, other alcohol consumption, HDI, SDI, and GDP on GBD. The fourth chapter contains results and a discussion of data analysis. The fifth chapter concludes with highlights and limitations of the research.

## Literature review

Alcohol consumption, as an independent variable plays a major role that affects the dependent variable of GBD. Consumption of alcohol leads to negative outcomes and is a prevalent worldwide main issue which significantly affects economic growth negatively. Income levels of countries have also affected alcohol consumption which is attributable to GBD. Many researchers have conducted studies regarding alcohol consumption and its attributable diseases taking into account countries, continents and the globe in the past but not for recent years.

Several social factors have been recognised as having an impact on alcohol consumption levels and patterns, as well as the severity of alcohol-related disorders in communities. Drinking alcohol is linked to a higher risk of mental and behavioural disorders, including alcoholism, significant non-communicable illnesses including liver cirrhosis, certain malignancies, cardiovascular diseases, and injuries from violence, traffic and conflicts. Below are continent-wide highlights based on the literature review.

### Empirical substantiations from Africa

In East Africa Middleton, Mmbaga [6] among males, a substantial part of Oesophageal Squamous Cell Carcinoma would be eliminated by abstention from or minimising alcoholic consumption. Leslie, Ahern [7] observed in South Africa variables that influence to reduce alcohol-related harms and dependency that offered new opportunities for improving population health. Furthermore WHO [1], among confirmed 39,000 deaths due to alcoholism in South Africa, the vast majority were men.

Coates, Kamanda [8] conducted major discrepancies in mortality rate for both communicable diseases and non-communicable diseases based on socio economic variables across seven health and demographic surveillance systems in Sub-Saharan Africa. It has been proved that the increasing use of any type of alcoholic beverage in African countries can lead to significant growth on GBD and impact spirits and alcoholic beverages more than others.

### Empirical substantiations from Asia

Sornpaisarn, Shield [9] proved that five middle-income countries in the South-East Asian region (Cambodia, Myanmar, Vietnam and Timor-Leste) globally stand on outright increase in adult alcohol per capita consumption. In India due to alcohol attributable health such as cancers, liver diseases and road traffic accidents, it is expected 593 million life years would be lost in four decades between the years 2011 and 2050 [10]. A few articles studied how middle-income South-East Asian countries contributed to alcohol related GBD. Nevertheless, further research studies need to be conducted in depth to identify how income levels vary with alcohol consumption and its attributable harm.

The risk of all types of cancer and cause of death in women and men were positively connected to heavy drinking [11]. The people in the habit of drinking alcohol had a notable increased risk of oral cancer with an odds ratio of 1.56 in the Hunan province of China [12]. Consumption of alcohol is more likely to develop non-communicable diseases among men having low socio economic status [13]. Findings of Jayathilaka, Selvanathan [14] on association of consumption of alcohol and poverty through an econometric model have demonstrated alcohol consumption, particularly illegal alcoholic beverages have a positively affect the poorer who are near to poverty line. Furthermore, it will be a value addition if researchers can incorporate socio demographic characteristics as variables including varying income levels regarding alcohol related GBD.

### Empirical substantiations from Europe

Poikolainen and Alanko [15] used Auto Regressive Distributed Lags model to find out log of non-spirit consumption and the log of spirit consumption and alcohol related deaths that were significantly co-integrated. In England in the year 1996, around 75,000 cases were recorded due to alcoholic liver disease, road traffic fatalities, suicide mainly affected alcohol attributable deaths among young people [16]. Rehm, Taylor [17] affirmed a high alcohol consumption in the WHO European region which was 12.1 litres of pure alcohol per capita and averagely higher than 100% upper considering the global consumption. Regarding the European continent, past research clearly indicated a significant impact of alcohol consumption on GBD. However, those researches were not up to date and lacked a clear explanation on how alcohol consumption and GBD vary, and which alcoholic beverages. Further, how HDI affected regarding alcohol attributable diseases was not under the scope of these studies.

Education, remunerated work, income, or a combination of these characteristics are commonly used to determine socioeconomic status. Studies by Kuntsche, Rehm [18] found a link between excessive drinking and educational attainment. Binge drinking is influenced by socioeconomic factors.

### Empirical substantiations from North America

In Canada, Ramstedt [19] observed a less geographical association in alcohol attributable cirrhosis. A peak in overall alcohol consumption occurred close to 1975 increased up alcohol related mortality and thereafter underwent a substantial decline until the 1990s as per Ramstedt [19]. The overall impression, nonetheless, is that, as a general rule, consumption and mortality are closely linked.

Over the past four decades, economic factors had limited direct effect on alcohol intake because these are Granger-caused by social or demographic changes in the United States [20]. The study on the United States based on data from 1999–2003 concluded long term and current income levels were linked to a higher likelihood of abstinence and human development, while current income would have indicated the link between long term income variance and adult alcohol consumption behaviours [21]. Cerdá, Johnson-Lawrence [21] further contained that while alcohol consumption may be more susceptible to short-term changes in the social environment, as a consequence, long-term income patterns may indirectly impact alcohol consumption.

### Empirical substantiations from Oceania

Selvanathan and Selvanathan [22] confirmed that in 1982, Australians consumed around 10 litres of pure alcohol per person which later fell closer to 7 litres per person. In contrast, beer consumption per capita has risen fourfold since 1975, peaking at 140 litres in 1975 and falling to a low of 93 litres in 1999; wine consumption has quadrupled, while spirits consumption has remained constant. The findings revealed that, while income and prices have a considerable impact on alcohol consumption patterns, the growing older population in Australia too is a significant contributor. However, in the Oceanian continent, further up to date and timely research studies should be developed on how alcohol consumption contributes to GBD. Socio demographic characteristics and income level have been considered as key variables regarding alcohol consumption and GBD.

### Empirical substantiations from global

Degenhardt, Charlson [5] revealed that alcohol and drug usage causes a substantial burden of diseases worldwide, varying considerably between countries and being strongly connected with social development. East Asia, South Asia, Eastern Europe, and Tropical Latin America have had the highest number of alcohol-attributable Disability Adjusted Life Years (DALYs), while East Asia, high income North America, South Asia, and Eastern Europe had the highest number of drug-attributable DALYs [5]. Justifying the alcohol attributable GBD, increased risk of many other health outcomes, including unintentional injuries and suicide, cancers and cirrhosis, represent a large portion of the GBD attributable to drug usage where chronic hepatitis C was a significant contributor [5].

With the use of the Multivariate Log-normal Mixture Poisson Distribution model, Manthey, Shield [2] discovered alcohol consumption per capita expanded globally from 59% in 1990 to 65% in 2017. It is expected to increase by 17% over the next 13 years, reaching 7.6 litres in 2030. In addition, European countries had a markedly drop in alcohol consumption compared to the several lower and upper middle-income countries in 2017 [2]. These findings align with the study conducted by Manthey, Shield [2]. Here, the findings confirmed that alcohol consumption in each continent and country varied along with their income levels.

Moreover, using econometric research, Zhang, Cao [23] discovered that approximately 819,435 deaths have occurred due to liver cancers in the world and alcohol consumption related to liver cancer mortality slightly dropped by 0.17% per year during their period of study. Moreover, after the year 2008, these researchers have noticed a significant rise in alcohol consumption and beverage types related to liver cancer mortality. Stein, Cruz-Lemini [24] employed the Multivariate model for studies in the developing world. Thus, it revealed that regular heavy drinking has a significant impact on the weight of alcohol in the cirrhosis burden at a population level.

Although several studies on the impact of alcohol consumption on GBD have been selected continents or regions, no comprehensive study encompassing all continents has been found in the literature. Also, no research deeply analyses the alcohol consumption, HDI, SDI, income as independent variables on dependent variable which is GBD globally and continent wise. In this context, further studies confirm that rising alcohol consumption is likely, which in turn, can intensify the overall burden of diseases in future.

## Methods

The data set contained observations about different cross sections across time, since the data analysis was done through panel regressions. Data from 2000 to 2019 for 177 countries and territories were collected under the variables GBD, beer, wine, spirit, other alcohol, HDI, SDI, and GDP. Data collection spanned for 51 African countries and territories, 47 Asian Countries, 37 European countries, 20 North American countries, 12 South American countries, and 10 Countries and territories in Australian continent. Data file used for the study is presented in S1 Appendix and the study has employed Stata, statistical software for the data analysis. Table 1 represents data sources and variables.

**Table 1:** Data sources and variables.

Variable	Definition	Source
*GBD*	Global Burden of Disease (DALYs per 100,000)	Institute for Health Metrics and Evaluation https://vizhub.healthdata.org/gbd-results/
*WINE*	Wine consumption (Recorded per capita consumption litres of pure alcohol)	World Health Organization Data https://www.who.int/data/gho/data/themes/topics/topic-details/GHO/levels-of-consumption World
*BEER*	Beer consumption (Recorded per capita consumption litres of pure alcohol)	
*SPIRIT*	Spirit consumption (Recorded per capita consumption litres of pure alcohol)	
*OTHER ALCOHOL*	Other alcoholic beverages consumption (Recorded per capita consumption litres of pure alcohol)	
*HDI*	Human Development Index (Composite indices)	United Nations Development Programme Data Centre https://www.hdr.undp.org/en/indicators/137506
*SDI*	Socio Demographic Index (Composite indices)	Institute for Health Metrics and Evaluation https://ghdx.healthdata.org/record/ihme-data/gbd-2019-socio-demographic-index-sdi-1950-2019
*GDP*	Income (GDP per capita current US $)	World Bank Open Data https://data.worldbank.org/indicator/NY.GDP.PCAP.CD

Source: Compiled by authors.

The main objective of this research is to investigate the impact of different types of alcohol varieties (wine, beer, spirit, and other types), total alcohol consumption, HDI, SDI and GDP on GBD. The linear model developed according to the past literature [25] is indicated below. The conceptual framework was developed (also aligns with the research proposal) whilst taking into account alcohol consumption (wine, beer, spirit, and other types), HDI, SDI, and GDP as the independent variables and the GBD as the dependent variable.


$${GBD}_{it}=\beta_{0}+\beta_{1}{WINE}_{it}+\beta_{2}{BEER}_{it}+\beta_{3}{SPIRIT}_{it}+\beta_{4}{OTHER\;ALCOHOL}_{it}+\beta_{5}{HDI}_{it}+\beta_{6}{SDI}_{it}+\beta_{7}{GDP}_{it}+\varepsilon_{it}$$ (1)

The above static linear model can be used to measure the impact of GBD in selected continents such as Africa, Asia, Europe, North America, Oceania, and South America for a long period like 20 years. Normally, the other alcohol types of category include illegal alcohol types and various types of alcohol unique to some countries and territories which do not fall under the categories such as wine, beer or spirits. As usual, HDI, SDI and GDP represent respective regions. Here, the relevant country is denoted by *i* and *t* shows the year taken into consideration. *ε_it_* shows the standard error of the above equation.

Among the independent variables in Eq 1, high correlation may cause the issue of multicollinearity particularly between HDI, SDI and GDP. Multicollinearity does not result in biased estimated coefficients. Multicollinearity or the small sample size may raise similar concerns, thereby, both tend to increase sampling variances. Increasing the sample size [26, 27] is the best way to minimise multicollinearity. With large sample sizes (> 100), normality assumption violation may not result in major issues [26, 28]. The present study used a large sample with 177 countries, which is likely to mitigate multicollinearity related concerns and normality assumptions and strengthen credibility of findings.

Since the data set have a variability and it changes across time with a country-specific impact, panel data approach is the most suitable model for data analysis. Three potential estimations models of balanced panel data regression were used namely Pooled Ordinary Least Square (POLS), Fixed Effects (FE) and Random Effects (RE) models [29]. To explore the impact of distribution GBD, study applied POLS model of panel data regression as shown in Eq 1. In panel regression analysis, time fixed effect captures the unobserved effects of variables that are constant across countries but can vary over time. Panel data analysis also suggests the application of the random effects model. The rationale is that the individual-specific country effect or variation across countries are assumed to be a random variable that is uncorrelated with the GBD. These three models will be used to compare the applicability of the desired model by using F testing, Lagrange multiplier testing and Hausman tests [29]. After obtaining the hypothesis test results of each test methods, the best model is selected following a sensitivity analysis. F test will be used to select the best model among POLS and FE. The Lagrange Multiplier’s test will be conducted with POLS and RE to select the model suitable for the next step. Hausman’s test will be performed for RE and FE to find the best model out of them.

All the variables were used under a standard approach. To reduce inconsistency of the size of variables, the constant factors applied for each variable is adapted accordingly with the standard approach. With the purpose of minimising the issue related to heteroscedasticity, data were changed into robust standard errors.

## Results

Averagely 1,365 of DALYs would be encountered a year globally due to alcohol related health conditions as per information sorted and data. This could be mainly addressed through eliminating alcohol consumption. According to Table 2 which illustrates summary descriptive statistics for the key variables, average GBD for the global is 1394.309 (DALYs per 100,000); highest value recorded for the European. As well as GBD, Europe records the highest spirit and beer consumption, which is 2.631 and 3.907 (Recorded per capita consumption litres of pure alcohol), respectively. Asian continent has the highest HDI even though it has high level of alcohol consumption. Furthermore, African continent reports the lowest averaged GDP per capita considered to all the continents where also notable lower SDI and HDI rates were reported in the continent.

**Table 2:** Summary descriptive statistics for the key variables.

Variables									
Countries		GBD (DALYs per 100,000)	Wine (Recorded per capita consumption litres of pure alcohol)	Beer (Recorded per capita consumption litres of pure alcohol)	Spirit (Recorded per capita consumption litres of pure alcohol)	Other Alcohol (Recorded per capita consumption litres of pure alcohol)	HDI (Composite indices)	SDI (Composite indices)	GDP (GDP per capita current US $)
**Global**	Obs.	3540	3518	3518	3518	3517	3485	3540	3523
	Mean	1365.71	0.980	1.886	1.435	0.545	0.680	0.584	11970.43
	SD	1021.72	1.520	1.770	1.537	1.470	0.161	0.188	17911.22
	Min.	73.80	0	0	0	0	0.262	0.088	111.93
	Max.	8926.97	6.680	8.62	8.810	11.110	0.957	0.929	154919.20
**Africa**	Obs.	1020	1020	1020	1020	1020	1007	1020	1011
	Mean	1191.17	0.32	1.076	0.416	1.245	0.512	0.402	2284.03
	SD	788.43	0.716	1.330	0.719	2.182	0.118	0.143	3124.89
	Min.	129.55	0	0	0	0	0.262	0.089	111.93
	Max.	3791.90	5.180	6.410	4.620	11.110	0.804	0.724	22942.61
**Asia**	Obs.	940	935	935	935	935	920	940	937
	Mean	1031.16	0.251	0.762	1.293	0.293	0.697	0.606	11243.60
	SD	1196.08	0.577	0.945	1.632	1.257	0.123	0.158	16805.38
	Min.	73.80	0	0	0	0	0.350	0.188	138.43
	Max.	8926.97	3.980	4.720	8.810	8.220	0.938	0.880	154919.20
**Europe**	Obs.	740	728	728	728	728	734	740	740
	Mean	2211.15	3.137	3.915	2.553	0.389	0.852	0.788	28585.59
	SD	1067.74	1.586	1.621	1.408	0.824	0.063	0.068	23870.15
	Min.	717.21	0.170	0.290	0.550	0	0.671	0.569	635.70
	Max.	5778.88	6.680	8.620	8.360	6.890	0.957	0.929	118823.60
**North America**	Obs.	400	395	395	395	395	393	400	400
	Mean	1298.39	0.508	2.484	2.585	0.097	0.727	0.621	11717.88
	SD	396.91	0.683	1.267	1.459	0.120	0.102	0.125	13442.41
	Min.	466.07	0.010	0.100	0.120	0	0.442	0.350	542.33
	Max.	6219.92	3.950	5.400	7.380	0.500	0.929	0.873	65279.53
**Oceania**	Obs.	200	200	200	200	200	195	200	200
	Mean	754.65	0.732	1.648	0.547	0.180	0.180	0.564	9834.07
	SD	339.22	1.276	1.432	0.515	0.382	0.140	0.155	15956.23
	Min.	304.84	0	0.030	0	0	0.450	0.328	491.83
	Max.	1353.37	3.870	5.070	2.070	1.260	0.944	0.840	68156.63
**South America**	Obs.	240	240	240	240	239	236	240	235
	Mean	1432.52	1.056	2.760	1.774	0.069	0.730	0.602	6468.50
	SD	441.66	1.485	1.036	1.067	0.155	0.057	0.057	4218.39
	Min.	761.98	0	0.850	0.300	0	0.616	0.474	904.23
	Max.	2664.97	5.320	6.850	6.570	0.890	0.851	0.759	18703.86

Note: SD represent Standard Deviation. Source: Authors’ calculations from Stata output.

**Fig 1:**
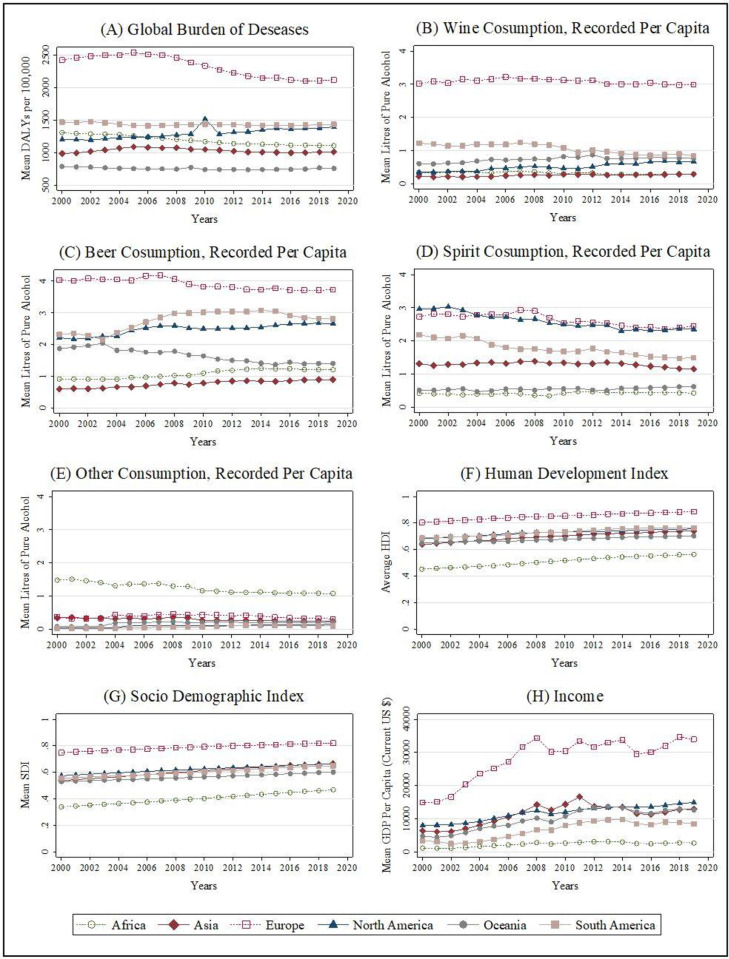
Continent wise averaged variables from 2000 to 2019. Source: Authors’ illustration based on the data.

Fig 1 illustrates mean variations of dependent and independent variables of this study between 2000 and 2019 at the continent level. Analysis of GBD attributable to alcohol reveals that European continent has a significant increased GBD attributable to alcohol than other continents where it also reveals higher intake of wine, beer and spirits among Europeans than other regions. Asian consumption of wine and beer is the lowest of all the continents while consumption of spirit states is much higher than Oceanian and African continents. Beer consumption of Oceanian countries which was higher than in Asia and Africa in early 20’s has significantly decreased later resulting in the similar decreasing trend in spirit consumption in South American continent countries within the same period. Consumption of other alcoholic beverages (Fig 1F) of all continents have low mean when compared to consumption of wine, beer and spirits. African continent countries with the lowest human development among all are having considerable amount of higher alcohol consumption trends (Fig 1A). European continent shows a higher amount of GBD attributable to alcohol in comparison to other continents where countries such as Ukraine, Belarus, Lithuania, Hungary and Estonia of European region are among the highest GBD. This trend has been decreasing in recent years, where the countries in the continent make stronger economies in the meantime. Compared to other continents, Oceania region records the lowest amount of GBD due to alcohol.

Graphs depicting means of wine consumption (Fig 1B), beer consumption (Fig 1C) and spirit consumption (Fig 1D) are presented below. Accordingly, European countries which have a higher standard of socio demographic development should pay more attention on policies and taxes to maintain decreased levels of their alcoholic beverage consumption.

Mean HDI graph (Fig 1F) and Mean SDI (Fig 1F) graph show how the key dimensions of human development and socio-demographic development vary across the world regions. Higher human development and socio-demographic development are estimated in other continents than in the European continent, as the latter comprises of strong economic nations. Though difference levels can be noted continent wise, the trend of development in human development dimensions and socio-demographic development could be seen within every continent through the years as there is no setback in development. Here, the North American, South American and Oceanian countries show similar human development trend that go hand in hand. Asian countries too fall under a similar category in terms of both human and socio-demographic development though some nations have low income levels with considerable inflation while several countries are strong with high economic conditions. African continent tends to have the lowest human development dimensions and socio-demographic development when compared to other continents.

Variations of mean income in terms of mean GDP per capita among continents worldwide are presented in the graph (Fig 1H). This displays strong economies of the European countries with the highest GDP values. Having several countries with strong economies in the North American and Oceanian continent resulted in higher GDP values throughout the period considered for the study, eventhough these are less than the GDP values in the European region. Though most countries in the Asian region have fragile economic conditions, it is indicated that several Asian economies hold strong and steady to that of North American and Oceanian continents. Same as in socio demograpic development and human development, African continent indicate low economic status and it is non growing nor shrinking in the periods shown. Furthermore, sensitivity analysis was conducted to explore the presence of any specification or multicorrelation issues of HDI, SDI and GDP. Detail results are presented in S2 Appendix and confirmed keeping those three variables together in the model would not lead to any considerable specification or mulicorrelation issues.

Results of panel data model specification tests for Eq (1) are presented in Table 3. Considering the findings of Poolability F test and Breusch-Pagan LM test for all the countries and continents reject the null hypothesis. Rejection of POLS model at a 1% significance level means that it is not the finest approach for current analysis. Thus, Hausmen test is employed to carry out the selection between FE and RE models. Test for the all countries, African, European, North American and South American continents has rejected the null hypothesis where RE model indicates that estimations result from FE model are more efficient than RE model. However, the tests on Asian and Oceanian could not reject the null hypothesis, thus indicated that estimation result of RE model is more efficient than FE. Thus, FE model could used for all countries, African, European, North American and South American continents, while Asia and Oceania continents, were continued with the RE model. Based on the test results, FE and RE models were used separately for the continents.

**Table 3:** Specification tests for panel data models.

Region	Tests		
	F test	LM Test	Hausman Test (Sigmamore)
	H_0_: POLS	H_0_: POLS	H_0_: Random Effect
	H_1_: Fixed Effect	H_1_: Random Effect	H_1_: Fixed Effect
All countries	193.66***	24249.19***	65.40***
Africa	259.71***	6652.89***	37.32***
Asia	197.85***	6380.54***	10.32
Europe	205.27***	4307.42***	44.40***
North America	23.00***	703.34***	14.14**
Oceania	170.72 ***	821.28***	9.20
South America	254.53 ***	959.09***	17.18***

Note: The symbols *, ** and *** represents 10%, 5% and 1% significance level, respectively.

Table 4 above illustrates the effect of wine, beer, spirit, other alcohol, HDI, SDI and GDP on GBD in a global and continent level, presenting the coefficients, standard errors and significance levels in a statistical summary. According to the overall R^2^ values on global and regional levels, over 40% of variation in GBD can be explained and fits the model for all countries and Asian and Oceanian regions. Over 20% of variation in GBD in the African and European regions fits the model where data explain less variations in GBD in North America and South America.

**Table 4:** Fixed effect and Random effect estimates.

Variables	All Countries		Africa		Asia		Europe		North America		Oceania		South America	
	*GBD*		*GBD*		*GBD*		*GBD*		*GBD*		*GBD*		*GBD*	
	FE	RE	FE	RE	FE	RE	FE	RE	FE	RE	FE	RE	FE	RE
***Wine***	121.17***	131.70***	97.11**	111.67**	217.15	216.66	98.99**	96.55**	89.91*	54.67	-11.53	9.14	-4.64	8.11
	(31.042)	(27.372)	(45.262)	(46.170)	(160.632)	(158.670)	(47.793)	(46.492)	(43.696)	(35.940)	(57.770)	(56.858)	(27.540)	(28.645)
***Beer***	115.14***	121.43***	59.09**	69.08**	194.80**	205.06**	100.41***	92.87***	6.68	-5.59	85.43***	90.52***	7.65	6.52
	(23.547)	(21.805)	(29.306)	(28.908)	(83.979)	(83.695)	(32.974)	(30.325)	(31.659)	(22.031)	(14.486)	(10.193)	(27.197)	(26.732)
***Spirit***	134.67***	145.77***	88.20***	94.65***	254.02**	258.42**	50.02	66.02*	-66.03	-61.13	68.78*	71.74**	2.61	6.85
	(50.419)	(49.826)	(19.977)	(21.444)	(112.697)	(112.688)	(40.991)	(37.125)	(68.837)	(51.127)	(30.441)	(29.729)	(16.753)	(15.956)
***Other alcohol***	51.17**	57.13**	60.70**	64.10**	21.94	24.22	170.20***	179.18***	-107.99	-44.91	10.45	9.60	-107.34	-78.12
	(23.856)	(24.456)	(25.746)	(25.854)	(27.672)	(28.341)	(48.266)	(47.637)	(178.165)	(139.015)	(36.474)	(35.973)	(69.986)	(74.003)
***HDI***	-2276.98***	-2419.22***	-3146.78***	-3268.23***	-1480.57	-1577.27	2777.44	2024.76	-979.83	-1927.64	-1375.32	-1480.45	-2459.76	-2739.63
	(672.810)	(681.333)	(982.271)	(994.016)	(1068.063)	(1093.286)	(2585.229)	(2274.519)	(1286.442)	(1573.660)	(1059.693)	(986.908)	(1829.585)	(1900.899)
***SDI***	1055.38**	1274.63***	1486.45*	1655.26*	598.852	658.256	- 6885.61**	-5978.63**	1517.39**	1519.866	1074.869	1255.909	1405.443	1691.222
	(483.449)	(489.891)	(874.193)	(892.444)	(492.412)	(524.073)	(3163.836)	(2830.745)	(533.882)	(961.838)	(1012.665)	(834.295)	(1534.027)	(1587.552)
***GDP***	-0.001*	-0.002*	-0.013	-0.014*	-0.001	-0.001	-0.002	-0.001	0.010**	0.009*	0.003*	0.003*	0.006	0.007
	(0.001)	(0.001)	(0.008)	(0.008)	(0.001)	(0.001)	(0.001)	(0.001)	(0.004)	(0.005)	(0.002)	(0.002)	(0.005)	(0.005)
***Constant***	1756.79***	1684.40***	2026.70***	1997.22***	1172.33***	1195.37**	4436.40***	4363.35***	1077.39	1801.50**	871.91*	818.04*	2334.99***	2339.98***
	(234.546)	(232.085)	(221.890)	(275.326)	(425.445)	(480.031)	(1004.807)	(1087.497)	(1019.322)	(892.444)	(452.493)	(459.754)	(701.560)	(692.448)
**N**	3453	3453	998	998	914	914	728	728	388	388	195	195	230	230
**No of Countries**	177	177	51	51	47	47	37	37	20	20	10	10	12	12
**No of years**	20	20	20	20	20	20	20	20	20	20	20	20	20	20
**R**^**2**^ **within**	0.205	0.205	0.352	0.351	0.396	0.396	0.388	0.385	0.067	0.060	0.330	0.327	0.108	0.106
**R**^**2**^ **Between**	0.540	0.541	0.313	0.365	0.567	0.570	0.242	0.318	0.009	0.001	0.657	0.665	0.112	0.286
**R**^**2**^ **Overall**	0.518	0.520	0.309	0.356	0.552	0.554	0.245	0.314	0.001	0.006	0.645	0.655	0.109	0.263

Note: The symbols *, ** and *** represents 10%, 5% and 1% significance level, respectively. Parentheses represent the robust standard error. FE and RE represent the Fixed effect and Random effect, respectively. N represent number of observations.

## Discussion

Statistical results for global context of both FE and RE panel data regression indicate that wine, beer, spirit, other alcohol and SDI have a positive influence on GBD at a significance level of 1%. Shield, Manthey [30] conducted a global level study for comparative risk assessment which identified the disproportionate effect of HDI on alcohol attributable burden. It confirms that HDI has a negative significant influence on GBD. Highly significant and positive coefficients of wine, beer and spirit conclude that irrespective of the geographical region, increased consumption of wine, beer, spirit can cause increased disease burden in a country. Similarly, Degenhardt, Charlson [5] have identified that alcohol could cause substantial disease burden globally under descriptive statistics where it varies between countries. Higher negative significant HDI coefficients indicate cause of higher human development impact to decrease disease burden levels.

Looking at the statistical results continent wise, GBD of African region is found to be highly significantly and positively influenced by the spirit consumption. With regard to countries with low HDI, particularly countries in sub-Saharan Africa with low HDI recorded indicate a higher burden of disease attributable to alcohol [30]. Comparably, this study shows negative influence of HDI in both FE and RE models which are highly significant at the 1% significance level on GBD. Compared to other variables in RE and FE estimates, consumption of wine, beer and other alcoholic beverages only have a considerable significant positive effect on GBD. Kuteesa, Seeley [31] have also supported this finding related to alcohol stating that despite the pattern and local context, harmful drinking is common among Africans. Further, this situation has led to adverse consequences where poverty and work environment considered as key drivers of alcohol consumption.

FE and RE estimates of Asian continent suggest beer and spirit has a positive and statistically significant effect on GBD. However, prevalence of alcohol dependence is quite low in Asian continent relatively to other continents [32]. However, according to analysis of Sornpaisarn, Shield [9], trends of five South-East Asian countries are among 12 countries with the most increasing alcohol consumption globally. Asia as a continent with more low and middle income countries and been in economic developments may have directly and indirectly impacted the increased alcohol consumption and increased alcohol attributed harm particularly in South-East Asian region; moreover, heavy episodic drinking is attributable to disease burden in Asia [9, 30]. Considerable number of countries in Asia consuming spirits and beer significantly impact GBD where other variables do not significantly impact GBD in the same region.

Alcoholic consumptions including beer and other alcoholic beverages indicate positive and highly significant effect, where wine consumption has a positive and considerable effect on GBD for European continent in both RE and FE estimates. According to the latter, hard liquor does not have a significant effect on alcohol attributable disease burden. In terms of alcohol attributable deaths, years of life lost and DALYs, Europe has the highest alcohol attributable burden of diseases compared to other regions [17]. Though the consumptions of alcoholic beverages in Europe have been decreasing over last decades, decrease in drivers on disease related burden have been caused due to advancement of social, economic, cultural and demographic determinants [33, 34]. According to the results, countries that have high development of socio demographic have a negative influence on burden of diseases. However, data of the study only explains variations of 24.5% and 31.4% of GBD through FE and RE estimates, respectively.

Estimation for North American continent of SDI from FE estimates and GDP from both FE and RE estimates variables are positive and significant towards GBD. As such, it could be explained that the development of sociodemographic environment and increase of GDP per capita have an effect on increasing GBD in North America. A study conducted by Degenhardt, Charlson [5] stated that North America is highly accounted for the drug use attributable DALYs while alcohol attributable DALYs is less. This means that there aren’t any significant impact of wine, beer, spirit and other alcohol on burden of disease in North America on GBD.

Oceanian continent only shows significant coefficients for beer consumption with a positive and significant influence towards GBD in both FE and RE estimates. Spirit consumption has a considerable effect on GBD. Study of Selvanathan and Selvanathan [22] answered for the estimates of higher significance through price elasticity. In Australia, beer price being inelastic and spirits being price elastic, where it held 60% of continent’s population. This study also concluded that when affordability of alcohol is increased, the consumption also increased. This explained why GDP considered as income is positively significant on GBD in both FE and RE estimates.

Based on the estimated models, coefficient of wine is highly significant for both RE and FE in global level taking African and European continent into consideration. Beer in global context have a highly significant positive influence on GBD for both RE and FE model in the European and Oceanian continents while considerably and positively significant on African and Asian continents. Spirits as a hard liquor have a highly significant coefficient in Africa on both RE and FE estimates and somewhat positively significant in Asian and Oceania continents, which have overall effect on significance in the global context. Moreover, the other alcohol has a significant effect on GBD at 5% in the global context considering its significance in African and European regions in both RE and FE models. HDI and SDI affect the GDB negatively and positively, respectively on both HDI and SDI, which have higher coefficient values together are highly significant on the effect on disease burden in the global context. Here, HDI of the African continent is significant in both RE and FE estimates. Higher significant effect of GDP on GBD have not been estimated on global context when considering other variables.

## Conclusion

Many previous empirical studies have shown that alcohol consumption and its misuse can have an unfavourable influence on the economic conditions, social, health and productivity exposure of communities. The present study is the first of its kind to be conducted in countries across all continents using the panel data analysis method. It focusses on the overall effect of HDI, SDI and GDP for income to measure how these variables impact GBD globally and the impact beyond.

The study further examines the percentage or weight of global consumption of beer, wine, spirit and other factors that contribute to alcohol consumption, both globally and continent wise. A Panel regression technique adopted in this research covered 177 countries and territories for a period of around 20 years from 2000 to 2019, applying the RE model with FE model after a Hausman test.

The results of this article further reveal the importance of the independent variables HDI, SDI, GDP, and alcohol consumption (wine, beer, spirit and other alcohol) affecting GBD. These conclusions led to both positive and negative outcomes as well as mixed outcomes. Statistical results for the global context of both FE and RE panel data regression indicate that in terms of wine, beer, spirit and other alcohol consumption, SDI has a positive and HDI has a negative influence on GBD at a significance level of 1%. Highly significant and positive coefficients of wine, beer, spirit and other alcohol conclude that irrespective of the geographical region, increased consumption of wine, beer, spirit and other alcohol can cause increased disease burden in a country.

Considering the impact of wine, beer, spirit and other consumption on GBD, the continent of Oceania have the greatest impact from beer consumption, while the continent of Africa has the greatest impact from the spirit and other alcohol consumption. For example, in the Oceanian continent, countries like New Zealand and Australia contribute to beer consumption on a large scale. FE and RE estimates of the Europe and the African continents suggest wine consumption has a positive significant effect on GBD. One of the species seen here is the SDI, which has the highest incidence of GBD in North America. Income does not have a major impact on the GBD, and the African continent has a negative significant impact on the HDI.

Perhaps in countries like Sri Lanka, income is not a variable that significantly impacts alcohol consumption and GBD. Another point is that in Sri Lanka, alcohol consumption can be seen mostly in terms of illicit liquor. This situation can be common for countries in the South Asian region. Hence, future studies can include more unique variables in terms of regions for a meaningful analysis.

This study has some limitations, as the missing data of 15 countries were dropped from the analysis. Periods of 2020 and 2021 were not considered since data was not available to be retrieved during the study period. Only the data available in databases were retrieved. The study employed HDI, SDI, GDP and global consumption of wine, beer, spirit and other alcohol types for independent variables as other related variables that might impact could not be considered due to lack of availability of reliable data sources. This study investigations were limited only to identify the unidirectional impact, but bio direction impacts may occur as well.

Alcohol as a whole, has been proven to be harmful to developed countries, especially in developing countries. Further studies confirm that it may increase its benefaction to the overall burden of disease in future. Moreover, previous researchers have estimated how the global alcohol consumption patterns varied country wise and globally. In this study, researchers aim to investigate the impact of global alcohol consumption on GBD while analysing the combined effect of HDI, SDI and GDP for the world regions continent wise. Further, by analysing these data, we expect to propose approaches that are beneficial and interventions to minimise and control alcohol consumption that are attributed to GBD.

## Supporting information

S1 Appendix Data file. (XLS)

S2 Appendix Sensitivity analysis. (DOCX)

## References

[1] WHO. Global Status Report on Alcohol and Health 2018. 2018.

[2] Manthey J, Shield KD, Rylett M, Hasan OS, Probst C, Rehm J. Global alcohol exposure between 1990 and 2017 and forecasts until 2030: a modelling study. The Lancet. 2019;393(10190):2493–502. doi: 10.1016/S0140-6736(18)32744-2 31076174

[3] IHME. Institute for Health Metrics and Evaluation 2021 [21st September 2021]. Available from: http://www.healthdata.org/.

[4] Rehm J, Imtiaz S. A narrative review of alcohol consumption as a risk factor for global burden of disease. Substance abuse treatment, prevention, and policy. 2016;11(1):1–12. doi: 10.1186/s13011-016-0081-2 27793173 PMC5084343

[5] Degenhardt L, Charlson F, Ferrari A, Santomauro D, Erskine H, Mantilla-Herrara A, et al. The global burden of disease attributable to alcohol and drug use in 195 countries and territories, 1990–2016: a systematic analysis for the Global Burden of Disease Study 2016. The Lancet Psychiatry. 2018;5(12):987–1012. doi: 10.1016/S2215-0366(18)30337-7 30392731 PMC6251968

[6] Middleton DR, Mmbaga BT, Menya D, Dzamalala C, Nyakunga-Maro G, Finch P, et al. Alcohol consumption and oesophageal squamous cell cancer risk in east Africa: findings from the large multicentre ESCCAPE case-control study in Kenya, Tanzania, and Malawi. The Lancet Global Health. 2022;10(2):e236–e45. doi: 10.1016/S2214-109X(21)00506-4 34921758 PMC8766315

[7] Leslie HH, Ahern J, Pettifor AE, Twine R, Kahn K, Gómez-Olivé FX, et al. Collective efficacy, alcohol outlet density, and young men’s alcohol use in rural South Africa. Health & place. 2015;34:190–8. doi: 10.1016/j.healthplace.2015.05.014 26071651 PMC4497916

[8] Coates MM, Kamanda M, Kintu A, Arikpo I, Chauque A, Mengesha MM, et al. A comparison of all-cause and cause-specific mortality by household socioeconomic status across seven INDEPTH network health and demographic surveillance systems in sub-Saharan Africa. Global health action. 2019;12(1):1608013. doi: 10.1080/16549716.2019.1608013 31092155 PMC6534200

[9] Sornpaisarn B, Shield K, Manthey J, Limmade Y, Low WY, Van Thang V, et al. Alcohol consumption and attributable harm in middle-income South-East Asian countries: Epidemiology and policy options. International Journal of Drug Policy. 2020;83:102856. doi: 10.1016/j.drugpo.2020.102856 32711336

[10] Jyani G, Prinja S, Ambekar A, Bahuguna P, Kumar R. Health impact and economic burden of alcohol consumption in India. International Journal of Drug Policy. 2019;69:34–42. doi: 10.1016/j.drugpo.2019.04.005 31055044

[11] Kim MK, Ko MJ, Han JT. Alcohol consumption and mortality from all-cause and cancers among 1.34 million Koreans: the results from the Korea national health insurance corporation’s health examinee cohort in 2000. Cancer Causes & Control. 2010;21(12):2295–302. doi: 10.1007/s10552-010-9656-9 20941640

[12] Hu Y, Zhong R, Li H, Zou Y. Effects of Betel Quid, Smoking and Alcohol on Oral Cancer Risk: A Case–Control Study in Hunan Province, China. Substance Use & Misuse. 2020;55(9):1501–8. doi: 10.1080/10826084.2020.1750031 32569534

[13] Kumari N, Salve PS. Substance use and non-communicable diseases in India: evidence from National Family Health Survey-4. Journal of Substance Use. 2021;26(1):30–5. doi: 10.1080/14659891.2020.176612

[14] Jayathilaka R, Selvanathan S, Bandaralage JS. Is there a link between alcohol consumption and the level of poverty? Applied Economics. 2016;48(22):2054–63. doi: 10.1080/00036846.2015.1114574

[15] Poikolainen K, Alanko T. Population alcohol consumption as a predictor of alcohol-specific deaths: a time-series analysis of aggregate data. Alcohol and Alcoholism. 2017;52(6):685–91. doi: 10.1093/alcalc/agx053 29016718

[16] Britton A, McPherson K. Mortality in England and Wales attributable to current alcohol consumption. Journal of Epidemiology & Community Health. 2001;55(6):383–8. doi: 10.1136/jech.55.6.383 11350993 PMC1731912

[17] Rehm J, Taylor B, Patra J. Volume of alcohol consumption, patterns of drinking and burden of disease in the European region 2002. Addiction. 2006;101(8):1086–95. doi: 10.1111/j.1360-0443.2006.01491.x 16869838

[18] Kuntsche E, Rehm J, Gmel G. Characteristics of binge drinkers in Europe. Social science & medicine. 2004;59(1):113–27. doi: 10.1016/j.socscimed.2003.10.009 15087148

[19] Ramstedt M. Alcohol consumption and alcohol-related mortality in Canada, 1950–2000. Canadian Journal of Public Health. 2004;95(2):121–6. doi: 10.1007/BF03405779 15074902 PMC6975701

[20] Brinkley GL. The causal relationship between socioeconomic factors and alcohol consumption: a Granger-causality time series analysis, 1950–1993. Journal of studies on alcohol. 1999;60(6):759–68. doi: 10.15288/jsa.1999.60.759 10606487

[21] Cerdá M, Johnson-Lawrence VD, Galea S. Lifetime income patterns and alcohol consumption: Investigating the association between long-and short-term income trajectories and drinking. Social science & medicine. 2011;73(8):1178–85. doi: 10.1016/j.socscimed.2011.07.025 21890256 PMC3185179

[22] Selvanathan E, Selvanathan S. Economic and demographic factors in Australian alcohol demand. Applied Economics. 2004;36(21):2405–17. doi: 10.1080/0003684042000280346

[23] Zhang G, Cao F, Shi L, Ma T, Zhang L. Contribution of high body mass index and alcohol use to liver cancer-related mortality: a study based on 195 countries or territories. Digestive and Liver Disease. 2020;52(2):221–31. doi: 10.1016/j.dld.2019.10.012 31744773

[24] Stein E, Cruz-Lemini M, Altamirano J, Ndugga N, Couper D, Abraldes JG, et al. Heavy daily alcohol intake at the population level predicts the weight of alcohol in cirrhosis burden worldwide. Journal of hepatology. 2016;65(5):998–1005. doi: 10.1016/j.jhep.2016.06.018 27392424

[25] Frimpong FA, Akwaa-Sekyi EK, Saladrigues R. Venture capital healthcare investments and health care sector growth: A panel data analysis of Europe. Borsa Istanbul Review. 2021. doi: 10.1016/j.bir.2021.06.008

[26] Wooldridge JM. Introductory Econometrics: A Modern Approach. 20 Channel Center Street, Boston, MA 02210, USA: Cengage; 2018.

[27] Greene WH. Econometric Analysis. Essex: Pearson Education Limited; 2020.

[28] Gujarati DN. Basic Econometrics: The McGraw−Hill Companies; 2004.

[29] Hill RC, Griffiths WE, Lim GC. Principles of econometrics: John Wiley & Sons; 2018.

[30] Shield K, Manthey J, Rylett M, Probst C, Wettlaufer A, Parry CD, et al. National, regional, and global burdens of disease from 2000 to 2016 attributable to alcohol use: a comparative risk assessment study. The Lancet Public Health. 2020;5(1):e51–e61. doi: 10.1016/S2468-2667(19)30231-2 31910980

[31] Kuteesa MO, Seeley J, Cook S, Webb EL. Multi-level experiences and determinants of alcohol misuse and illicit drug use among occupational groups at high-risk of HIV in sub-Saharan Africa: A thematic synthesis of qualitative findings. Global public health. 2020;15(5):715–33. doi: 10.1080/17441692.2019.1679216 31640453 PMC7175470

[32] Chen C-C, Yin S-J. Alcohol abuse and related factors in Asia. International Review of Psychiatry. 2008;20(5):425–33. doi: 10.1080/09540260802344075 19012127

[33] Voller F, Maccari F, Pepe P, Allamani A. Changing trends in European alcoholic beverage drinking: selected social, demographic, economic factors, drinking’s related harms, and prevention control policies between the 1960s and 2000s. Substance use & misuse. 2014;49(12):1515–30. doi: 10.3109/10826084.2014.914374 25099313

[34] Allamani A, Voller F, Decarli A, Casotto V, Pantzer K, Anderson P, et al. Contextual determinants of alcohol consumption changes and preventive alcohol policies: a 12-country European study in progress. Substance Use & Misuse. 2011;46(10):1288–303. doi: 10.3109/10826084.2011.572942 21692604

