# Supplementary material for: Alcohol brings burdens: A global and continent wise study on alcohol consumption and global burden of diseases
Source: PLoS One. 2022 Jul 28;17(7):e0270998. doi: 10.1371/journal.pone.0270998 (PMC9333294; doi:10.1371/journal.pone.0270998)
Supplement: S2 Appendix — (DOCX) [file pone.0270998.s002.docx]

**S2 Appendix. Sensitivity Analysis.**

**Table S1: Fixed effect and random effect estimates after dropping SDI**

| **Variables** | **All Countries** | | **Africa** | | **Asia** | | **Europe** | | **North America** | | **Oceania** | | **South America** | |
| --- | --- | --- | --- | --- | --- | --- | --- | --- | --- | --- | --- | --- | --- | --- |
|  | ***GBD*** | | ***GBD*** | | ***GBD*** | | ***GBD*** | | ***GBD*** | | ***GBD*** | | ***GBD*** | |
|  | **FE** | **RE** | **FE** | **RE** | **FE** | **RE** | **FE** | **RE** | **FE** | **RE** | **FE** | **RE** | **FE** | **RE** |
| ***Wine*** | 114.59*** | 126.37*** | 55.63 | 67.00* | 209.71 | 210.21 | 105.50** | 100.67** | 110.55*** | 72.30** | -4.81 | 13.69 | -11.85 | 2.727 |
|  | (30.284) | (26.917) | (33.643) | (35.135) | (159.235) | (157.191) | (46.038) | (45.57) | (38.607) | (36.480) | (63.110) | (60.521) | (31.191) | (32.384) |
| ***Beer*** | 120.05*** | 127.77*** | 69.60** | 80.41*** | 194.19** | 203.97** | 112.11*** | 100.37*** | 8.85 | -8.86 | 78.67*** | 83.16*** | 12.17 | 11.174 |
|  | (22.792) | (20.825) | (29.973) | (29.601) | (84.111) | (83.767) | (28.696) | (26.54) | (33.149) | (22.648) | (15.499) | (12.322) | (28.236) | (27.729) |
| ***Spirit*** | 130.34** | 140.84*** | 73.55*** | 78.12*** | 253.11** | 257.43** | 53.34 | 69.29** | -79.46 | -61.97 | 66.41* | 68.73** | -3.98 | -0.149 |
|  | (50.088) | (49.786) | (16.623) | (18.114) | (112.766) | (112.920) | (36.257) | (33.34) | (66.014) | (50.702) | (33.896) | (33.452) | (15.624) | (15.792) |
| ***Other alcohol*** | 48.18** | 53.51** | 53.07** | 55.52** | 21.38 | 23.77 | 159.34*** | 172.91*** | -122.98 | -19.70 | 2.23 | 1.17 | -127.64 | -95.17 |
|  | (23.691) | (24.370) | (25.967) | (26.240) | (27.111) | (27.820) | (53.580) | (52.05) | (177.947) | (123.617) | (40.286) | (39.462) | (84.891) | (89.703) |
| ***HDI*** | -1363.47*** | -1290.77*** | -1997.84*** | -1980.93*** | -922.36 | -954.99 | -2961.98*** | -2960.71*** | 481.60 | -405.60 | -456.42 | -384.26 | -1102.14 | -1100.03 |
|  | (360.044) | (333.881) | (496.819) | (487.044) | (739.007) | (707.351) | (991.423) | (999.94) | (1321.533) | (1081.574) | (653.325) | (619.675) | (1029.066) | (1021.028) |
| ***GDP*** | -0.001 | -0.001* | -0.008 | -0.008 | -0.001 | -0.001 | -0.001 | -0.001 | 0.011** | 0.01** | 0.003* | 0.003* | 0.005 | 0.005 |
|  | (0.001) | (0.001) | (0.007) | (0.007) | (0.001) | (0.001) | (0.001) | (0.001) | (0.004) | (0.004) | (0.002) | (0.002) | (0.005) | (0.005) |
| ***Constant*** | 1755.44*** | 1660.96*** | 2044.43*** | 2012.53*** | 1149.33*** | 1163.25** | 3809.82*** | 3838.12*** | 963.86 | 1624.01* | 867.81* | 797.93* | 2204.17*** | 2172.32*** |
|  | (235.751) | (231.738) | ( 230.498) | (291.802) | (418.430) | (469.639) | (886.054) | (964.40) | (1007.059) | (851.533) | (436.804) | (452.216) | (677.409) | (656.536) |
| **N** | 3,453 | 3,453 | 998 | 998 | 914 | 914 | 728 | 728 | 388 | 388 | 195 | 195 | 230 | 230 |
| **No of Countries** | 177 | 177 | 51 | 51 | 47 | 47 | 37 | 37 | 20 | 20 | 10 | 10 | 12 | 12 |
| **No of years** | 20 | 20 | 20 | 20 | 20 | 20 | 20 | 20 | 20 | 20 | 20 | 20 | 20 | 20 |
| **R^2^ within** | 0.198 | 0.198 | 0.322 | 0.321 | 0.394 | 0.394 | 0.353 | 0.351 | 0.063 | 0.056 | 0.308 | 0.305 | 0.083 | 0.080 |
| **R^2^ Between** | 0.506 | 0.511 | 0.143 | 0.176 | 0.565 | 0.567 | 0.472 | 0.547 | 0.014 | 0.000 | 0.579 | 0.595 | 0.001 | 0.017 |
| **R^2^ Overall** | 0.485 | 0.491 | 0.151 | 0.183 | 0.550 | 0.551 | 0.426 | 0.495 | 0.002 | 0.002 | 0.562 | 0.582 | 0.000 | 0.019 |

Note: The symbols *, ** and *** represents 10%, 5% and 1% significance level, respectively. Parentheses represent the robust standard error. FE and RE represent the Fixed effect and Random effect, respectively. N represent number of observations.

**Table S2: Fixed effect and random effect estimates after dropping HDI**

| **Variables** | **All Countries** | | **Africa** | | **Asia** | | **Europe** | | **North America** | | **Oceania** | | **South America** | |
| --- | --- | --- | --- | --- | --- | --- | --- | --- | --- | --- | --- | --- | --- | --- |
|  | ***GBD*** | | ***GBD*** | | ***GBD*** | | ***GBD*** | | ***GBD*** | | ***GBD*** | | ***GBD*** | |
|  | **FE** | **RE** | **FE** | **RE** | **FE** | **RE** | **FE** | **RE** | **FE** | **RE** | **FE** | **RE** | **FE** | **RE** |
| ***Wine*** | 107.28*** | 117.86*** | 4.53 | 22.14 | 165.51 | 166.08 | 102.29* | 98.592** | 103.54** | 83.11** | -2.77 | 87.56 | -11.02 | 1.437 |
|  | (30.004) | (117.856) | (41.556) | (41.260) | (131.18) | (130.196) | (45.747) | (45.199) | (47.218) | (39.114) | (65.479) | (62.933) | (28.576) | (29.900) |
| ***Beer*** | 111.33*** | 117.22*** | 55.132* | 67.93** | 179.70** | 188.47** | 105.53** | 96.11*** | 6.71 | 14.399 | 83.983*** | 87.56*** | 5.35 | 3.20 |
|  | (22.066) | (20.320) | (31.126) | (30.352) | (80.787) | (79.831) | (31.693) | (29.433) | (29.460) | (29.293) | (16.496) | (12.842) | (24.891) | (24.400) |
| ***Spirit*** | 127.53** | 138.07*** | 54.89*** | 61.58*** | 251.96** | 256.54** | 50.29 | 65.61* | -66.97 | -58.36 | 74.54* | 74.98** | 1.74 | 6.94 |
|  | (50.919) | (50.509) | (19.146) | (20.862) | (114.637) | (114.970) | (39.295) | (36.226) | (68.167) | (47.546) | (33.592) | (32.049) | (18.954) | (18.350) |
| ***Other alcohol*** | 50.71** | 56.61** | 51.96* | 55.83* | 22.93 | 25.214 | 163.97** | 175.27*** | -102.99 | -4.76 | 12.093 | 11.54 | -125.08* | -97.47 |
|  | (24.056) | (24.727) | (30.503) | (30.696) | (25.165) | (25.695) | (47.253) | (46.783) | (164.095) | (125.811) | (38.476) | (36.860) | (67.208) | (77.434) |
| ***SDI*** | -774.02*** | -668.57*** | -1257.69*** | -1170.06*** | -376.87 | 386.36 | -3928.54** | -3852.02*** | 795.51 | 136.54 | -102.33 | -34.89 | -439.50 | -354.72 |
|  | (254.884) | (223.889) | (410.893) | (386.822) | (435.063) | (397.729) | (1201.466) | (1204.851) | (1065.456) | (831.566) | (696.353) | (616.944) | (920.469) | (906.304) |
| ***GDP*** | -0.002** | -0.002** | -0.014* | -0.015* | -0.001 | -0.001 | -0.001 | -0.002 | 0.009** | 0.007 | 0.003 | 0.003* | .001 | 0.001 |
|  | (0.001) | (0.001) | (0.008) | (0.008) | (0 .001) | (0.001) | (0.001) | (0.001) | (0.004) | (0.005) | (0.002) | (0.002) | (0.004) | (0.004) |
| ***Constant*** | 1315.64*** | 1215.86*** | 1582.83*** | 1522.12*** | 765.61*** | 756.05*** | 4420.084*** | 4378.41*** | 812.60 | 1271.59** | 602.98 | 550.32 | 1694.27*** | 1620.88*** |
|  | (167.183) | (157.310) | (159.211) | (205.306) | (230.918) | (261.05) | (1014.403) | (1097.606) | (732.204) | (582.447) | (392.575) | (376.609) | (500.964) | (464.307) |
| **N** | 3,453 | 3,453 | 998 | 998 | 914 | 914 | 728 | 728 | 388 | 388 | 195 | 195 | 230 | 230 |
| **No of Countries** | 177 | 177 | 51 | 51 | 47 | 47 | 37 | 37 | 20 | 20 | 10 | 10 | 12 | 12 |
| **No of years** | 20 | 20 | 20 | 20 | 20 | 20 | 20 | 20 | 20 | 20 | 20 | 20 | 20 | 20 |
| **R^2^ within** | 0.175 | 0.175 | 0.202 | 0.200 | 0.382 | 0.382 | 0.380 | 0.379 | 0.074 | 0.069 | 0.296 | 0.295 | 0.0362 | 0.033 |
| **R^2^ Between** | 0.494 | 0.497 | 0.047 | 0.093 | 0.559 | 0.561 | 0.350 | 0.405 | 0.017 | 0.010 | 0.575 | 0.585 | 0.0272 | 0.001 |
| **R^2^ Overall** | 0.471 | 0.475 | 0.054 | 0.097 | 0.541 | 0.542 | 0.335 | 0.385 | 0.003 | 0.001 | 0.564 | 0.575 | 0.0213 | 0.002 |

Note: The symbols *, ** and *** represents 10%, 5% and 1% significance level, respectively. Parentheses represent the robust standard error. FE and RE represent the Fixed effect and Random effect, respectively. N represent number of observations.

**Table S3: Fixed effect and random effect estimates after dropping GDP**

| **Variables** | **All Countries** | | **Africa** | | **Asia** | | **Europe** | | **North America** | | **Oceania** | | **South America** | |
| --- | --- | --- | --- | --- | --- | --- | --- | --- | --- | --- | --- | --- | --- | --- |
|  | ***GBD*** | | ***GBD*** | | ***GBD*** | | ***GBD*** | | ***GBD*** | | ***GBD*** | | ***GBD*** | |
|  | **FE** | **RE** | **FE** | **RE** | **FE** | **RE** | **FE** | **RE** | **FE** | **RE** | **FE** | **RE** | **FE** | **RE** |
| ***Wine*** | 119.55*** | 129.46*** | 104.39** | 117.63** | 215.24 | 214.80 | 100.03** | 97.42** | 98.23* | 74.95* | 21.47 | 35.52 | -15.68 | -1.37 |
|  | (31.172) | (27.691) | (48.056) | (49.240) | (159.698) | (157.828) | (47.411) | (46.252) | (49.660) | (43.717) | (60.165) | (62.131) | (23.191) | (25.289) |
| ***Beer*** | 108.57*** | 114.77*** | 51.61* | 60.23** | 195.18** | 205.15** | 105.65*** | 98.68*** | 9.49 | -1.06 | 77.57*** | 81.40*** | 19.75 | 20.04 |
|  | (23.761) | (22.485) | (30.092) | (30.339) | (84.109) | (83.832) | (32.820) | (30.428) | (34.093) | (28.152) | (17.920) | (14.196) | (14.382) | (14.426) |
| ***Spirit*** | 135.17*** | 145.85*** | 79.76*** | 85.18*** | 254.77** | 259.14** | 49.38 | 65.46* | -54.93 | -57.18 | 66.14* | 68.24** | 4.48 | 9.62 |
|  | (50.470) | (49.932) | (16.170) | (16.970) | (112.868) | (112.883) | (41.414) | (37.780) | (64.670) | (48.665) | (31.827) | (30.890) | (17.999) | (17.037) |
| ***Other alcohol*** | 50.43** | 55.86** | 58.99** | 62.11** | 22.05 | 24.24 | 164.46*** | 172.63*** | -686.65 | -28.26 | 81.22 | 77.25 | -121.68 | -87.12 |
|  | (23.693) | (24.208) | (25.291) | (25.367) | (27.611) | (28.248) | (48.671) | (47.769) | (171.313) | (132.319) | (78.976) | (82.383) | (73.950) | (83.546) |
| ***HDI*** | -2335.93*** | -2476.31*** | -3145.69*** | -3256.72*** | -1482.25 | -1576.87 | 2348.78 | 1559.69 | -686.65 | -1579.75 | -1312.25 | -1400.54 | -1764.72 | -1984.12 |
|  | (666.730) | (674.753) | (965.928) | (976.195) | (1065.073) | (1088.931) | (2453.263) | (2165.244) | (1236.064) | (1569.297) | (1036.372) | (990.513) | (1766.069) | (1832.906) |
| ***SDI*** | 1056.15** | 1255.60** | 1308.55 | 1454.11* | 554.98 | 606.69 | -6759.87** | -5906.83** | 1926.77*** | 1855.92** | 1159.30 | 1289.75 | 1073.23 | 1357.56 |
|  | (485.659) | (490.767) | (811.600) | (824.002) | (484.453) | (513.371) | (3138.156) | (2810.749) | (493.114) | (917.556) | (983.337) | (852.314) | (1476.858) | (1525.353) |
| ***Constant*** | 1793.17*** | 1730.55*** | 2076.80*** | 2053.23*** | 1188.41*** | 1214.22** | 4631.97*** | 4620.04*** | 682.20 | 1408.49* | 791.65 | 761.40* | 2040.80*** | 2000.07*** |
|  | (235.882) | (234.685) | (228.947) | (284.879) | (432.319) | (487.865) | (962.363) | (1039.483) | (932.996) | (851.089) | (444.560) | (457.633) | (574.893) | (578.297) |
| **N** | 3,453 | 3,453 | 998 | 998 | 914 | 914 | 728 | 728 | 388 | 388 | 195 | 195 | 230 | 230 |
| **No of Countries** | 177 | 177 | 51 | 51 | 47 | 47 | 37 | 37 | 20 | 20 | 10 | 10 | 12 | 12 |
| **No of years** | 20 | 20 | 20 | 20 | 20 | 20 | 20 | 20 | 20 | 20 | 20 | 20 | 20 | 20 |
| **R^2^ within** | 0.201 | 0.200 | 0.344 | 0.343 | 0.395 | 0.396 | 0.385 | 0.383 | 0.059 | 0.053 | 0.287 | 0.286 | 0.095 | 0.091 |
| **R^2^ Between** | 0.532 | 0.530 | 0.290 | 0.335 | 0.564 | 0.566 | 0.229 | 0.307 | 0.050 | 0.033 | 0.669 | 0.671 | 0.020 | 0.132 |
| **R^2^ Overall** | 0.509 | 0.509 | 0.287 | 0.328 | 0.549 | 0.551 | 0.233 | 0.301 | 0.014 | 0.005 | 0.657 | 0.660 | 0.022 | 0.124 |

Note: The symbols *, ** and *** represents 10%, 5% and 1% significance level, respectively. Parentheses represent the robust standard error. FE and RE represent the Fixed effect and Random effect, respectively. N represent number of observations.
